# Supplementary material for: Broad-Spectrum Inhibition of Respiratory Virus Infection by MicroRNA Mimics Targeting p38 MAPK Signaling
Source: Mol Ther Nucleic Acids. 2017 Apr 6;7:256–66. doi: 10.1016/j.omtn.2017.03.008 (PMC5415959; doi:10.1016/j.omtn.2017.03.008)
Supplement: Document S1. Figure S1 and Table S1 [file mmc1.pdf]

OMTN, Volume 7

Supplemental Information

Broad-Spectrum Inhibition of Respiratory Virus Infection by MicroRNA Mimics Targeting p38 MAPK Signaling

Jana L. McCaskill, Sarah Ressel, Andreas Alber, Jane Redford, Ultan F. Power, Jürgen Schwarze, Bernadette M. Dutia, and Amy H. Buck

Supplementary Table 1. Validated signalling pathway markers analysed via RPPA

Antibody	Supplier	Reference Number	Raised	Dilution
IKK alpha/beta P Ser176/Ser177	Cell Signaling Technologies	2078	rabbit	250
Bim	Epitomics	1036	rabbit	250
ErbB-2/Her2/EGFR P Tyr1248/	Cell Signaling Technologies	2244	rabbit	250
NFkB p65 Ser536	Cell Signaling Technologies	3033	rabbit	250
PLC-gamma1 P Tyr783	Cell Signaling Technologies	2821	rabbit	250
AMPK alpha	Cell Signaling Technologies	2532	rabbit	250
c-Jun N-term	Epitomics	1254-1	rabbit	250
CREB	Cell Signaling Technologies	9197	rabbit	250
Bad P Ser112	Cell Signaling Technologies	9291	rabbit	500
CrkL P Tyr207	Cell Signaling Technologies	3181	rabbit	500
FLT3 P Tyr591 P Tyr591	Cell Signaling Technologies	3461	rabbit	500
JAK1	Cell Signaling Technologies	3332	rabbit	500
MNK1 (MKNK) P Thr197,Thr20	Cell Signaling Technologies	2111	rabbit	500
p44/42 MAPK (ERK1/2)	Cell Signaling Technologies	9102	rabbit	500
p44/42 MAPK (ERK1/2) P Thr2	Cell Signaling Technologies	4370	rabbit	500
Src	Cell Signaling Technologies	2109	rabbit	500
Akt	Cell Signaling Technologies	9272	rabbit	500
Akt P Ser473	Cell Signaling Technologies	4060	rabbit	500
4E-BP1 P Ser65	Cell Signaling Technologies	9451	rabbit	500
beta-Catenin	Cell Signaling Technologies	9562	rabbit	500
SAPK/JNK	Cell Signaling Technologies	9258	rabbit	500
GSK-3-alpha/beta P Ser21/Ser9	Cell Signaling Technologies	9331	rabbit	500
p53 P Ser15	Cell Signaling Technologies	9284	rabbit	500
p38 MAPK PThr180,Tyr182	Cell Signaling Technologies	9211	rabbit	500
p38 MAPK	Cell Signaling Technologies	9212	rabbit	500
SAPK/JNK P Thr183,Tyr185	Cell Signaling Technologies	4668	rabbit	500
Rb P Ser780	Cell Signaling Technologies	9307	rabbit	500
Stat1 P Tyr701	Cell Signaling Technologies	9171	rabbit	500
Src (family) P Tyr416	Cell Signaling Technologies	2101	rabbit	500
Smad2/3 P Ser465/Ser423,Ser467	Cell Signaling Technologies	9510	rabbit	500
Cyclin D1 P Thr286	Cell Signaling Technologies	3300	rabbit	500
Bcl-2	Epitomics	1017-1	rabbit	500
Bid	Epitomics	1008	rabbit	500
Bim P Ser69	Cell Signaling Technologies	4585	rabbit	500
GSK-3-beta	Cell Signaling Technologies	9315	rabbit	500
PLC-gamma1	Cell Signaling Technologies	2822	rabbit	500
MAPKAPK-2	Epitomics	1497-1	rabbit	500
Stat3	Cell Signaling Technologies	9132	rabbit	500
XIAP	Cell Signaling Technologies	2045	rabbit	500
FRA1 (R20)	Santa Cruz	sc-605	rabbit	500
AMPK alpha P Thr172	Cell Signaling Technologies	2535	rabbit	500
IGF1R B	Cell Signaling Technologies	3027	rabbit	500
PKC (pan) P Ser660 (beta-2)	Cell Signaling Technologies	9371	rabbit	1000

PKC substrate P (R/K)X(S*)(Hyd)	Cell Signaling Technologies	2261	rabbit	1000
ErbB-1/EGFR	Cell Signaling Technologies	2232	rabbit	1000
EGFR P Tyr1173	Cell Signaling Technologies	4407	rabbit	1000
beta-Catenin P Ser33,Ser37,Thr41	Cell Signaling Technologies	9561	rabbit	1000
Bcl-x	Epitomics	1018	rabbit	1000
NFkB p105/p50	GeneTex	GTX110585	rabbit	1000
p21 CIP/WAF1 p Thr145	Santa Cruz	20220-R	rabbit	1000
JAK1 P Tyr1022,Thr1023	Invitrogen (Biosource)	44-422G	rabbit	5000
beta-Tubulin	Abcam	ab6046	rabbit	5000
Prohibitin	Santa Cruz	sc-28259	rabbit	5000
p53	Cell Signaling Technologies	9282	rabbit	10000
Histone H2A.X P Ser139	Millipore (Upstate)	05-636	mouse IgG1	250
CrkL	Cell Signaling Technologies	3182	mouse IgG1	500
Stat1	Cell Signaling Technologies	9176	mouse IgG1	500
Cyclin D1	Cell Signaling Technologies	2926	mouse IgG2a	500
PKR	Santa Cruz	sc-6282	mouse IgG2a	500
GAPDH	Abcam	ab9484	mouse IgG2b	2000
rabbit			rabbit	250
mouse IgG1			mouse IgG1	250
mouse IgG2a			mouse IgG2a	500
mouse IgG2b			mouse IgG2b	500

Supplementary Table 2. Mean Referenced Fluorescence Intensity (RFI value) normalised to prohibitin housekeeping gene

Excel file supplied

Supplementary Table 3. Infected sample RFI values normalised to prohibitin and analysed for expression increase or decrease of 25% when compared to the uninfected control average.

Excel file supplied

Supplementary Figure 1.

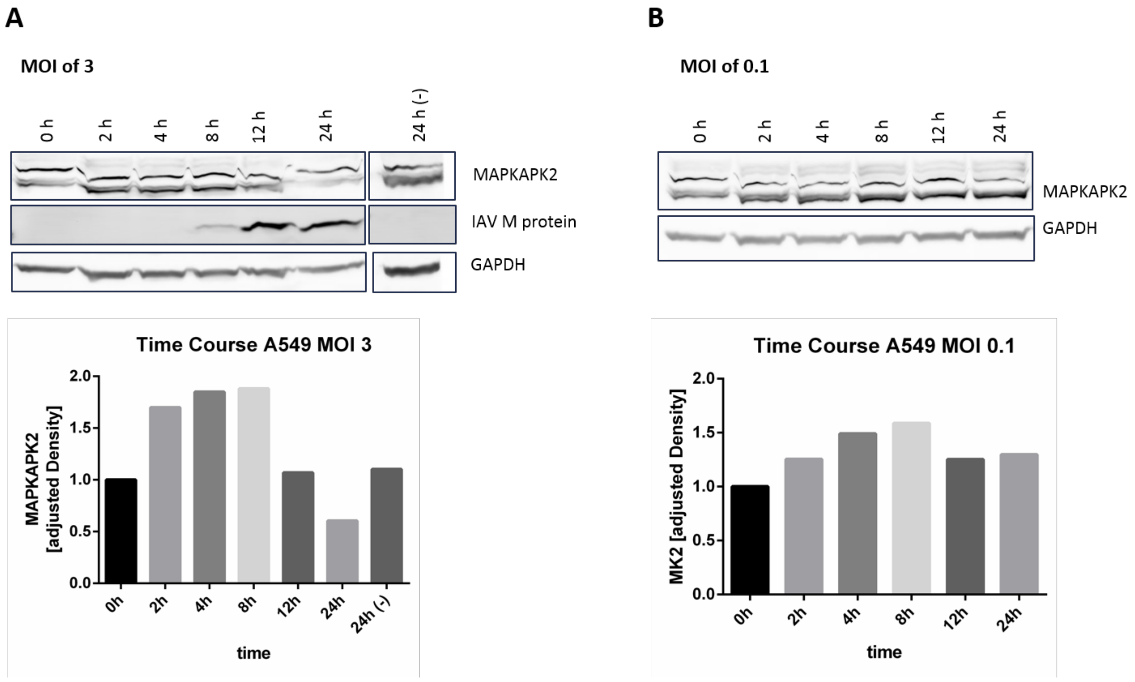

Supplementary Figure 1. Time course of MK2 expression during IAV infection. A549 cells were infected with IAV H1N1 WSN at an **A)** MOI of 3 or a **B)** MOI of 0.1. Western blot analysis and the corresponding band density analysis are displayed for each MOI. Quantitative analysis of MK2 was normalised to the GAPDH loading control and subsequently compared to uninfected sample (0hrs).
