# Supplementary material for: Broad-Spectrum Inhibition of Respiratory Virus Infection by MicroRNA Mimics Targeting p38 MAPK Signaling
Source: Mol Ther Nucleic Acids. 2017 Apr 6;7:256–66. doi: 10.1016/j.omtn.2017.03.008 (PMC5415959; doi:10.1016/j.omtn.2017.03.008)
Supplement: Document S2. Article plus Supplemental Information [file mmc4.pdf]

Molecular Therapy
Nucleic Acids
Original Article

Broad-Spectrum Inhibition of Respiratory Virus Infection by MicroRNA Mimics Targeting p38 MAPK Signaling

Jana L. McCaskill,[1] Sarah Ressel,[1] Andreas Alber,[2] Jane Redford,[1] Ultan F. Power,[3] Jürgen Schwarze,[4] Bernadette M. Dutia,[2] and Amy H. Buck[1]

[1]Institute of Immunology and Infection Research and Centre for Immunity, Infection and Evolution, School of Biological Sciences, University of Edinburgh, Edinburgh EH9 3FL, UK; [2]The Roslin Institute and Royal (Dick) School of Veterinary Studies, University of Edinburgh, Easter Bush, Edinburgh EH25 9RG, UK; [3]Centre for Experimental Medicine, School of Medicine, Dentistry and Biomedical Sciences, Queen's University Belfast, Belfast BT9 7BL, UK; [4]MRC-Centre for Inflammation Research, University of Edinburgh, The Queens Medical Research Institute, Edinburgh EH16 4TJ, UK

The majority of antiviral therapeutics target conserved viral proteins, however, this approach confers selective pressure on the virus and increases the probability of antiviral drug resistance. An alternative therapeutic strategy is to target the host-encoded factors that are required for virus infection, thus minimizing the opportunity for viral mutations that escape drug activity. MicroRNAs (miRNAs) are small noncoding RNAs that play diverse roles in normal and disease biology, and they generally operate through the post-transcriptional regulation of mRNA targets. We have previously identified cellular miRNAs that have antiviral activity against a broad range of herpesvirus infections, and here we extend the antiviral profile of a number of these miRNAs against influenza and respiratory syncytial virus. From these screening experiments, we identified broad-spectrum antiviral miRNAs that caused >75% viral suppression in all strains tested, and we examined their mechanism of action using reverse-phase protein array analysis. Targets of lead candidates, miR-124, miR-24, and miR-744, were identified within the p38 mitogen-activated protein kinase (MAPK) signaling pathway, and this work identified MAPK-activated protein kinase 2 as a broad-spectrum antiviral target required for both influenza and respiratory syncytial virus (RSV) infection.

INTRODUCTION

Since the discovery of microRNAs (miRNAs) in *Caenorhabditis elegans* in 1993, these molecules have been shown to play many important roles in stress and disease, including virus infection.[1] The miRNAs are small noncoding RNAs that normally bind to short regions of sequence similarity in mRNA targets to inhibit translation.[2] Emerging non-canonical functions of miRNAs have also been demonstrated, and multiple viruses have evolved to exploit the activity of host miRNAs for use in their life cycles. For example, hepatitis C virus encodes binding sites for liver-specific miR-122 to stabilize the viral genome, stimulate viral translation within the liver, and prevent the induction of innate immune responses.[3–5] In addition, Eastern equine encephalitis virus has been shown to encode a myeloid-specific miRNA-binding site in its genome to limit replication and, thereby, suppress innate immune induction in myeloid cells.[6] The therapeutic capacity of miRNA manipulation in viral infection has largely been explored in the context of blocking the interactions between a host miRNA and a viral sequence. However, in several cases it has been shown that viruses can also encode in their genomes inhibitors against specific host miRNAs, highlighting the natural antiviral properties of some members of this class of molecule.[7]

The use of miRNAs to target host factors that are utilized by viruses to promote infection and virus replication is a developing antiviral strategy, as it is hypothesized to overcome the selective pressure and subsequent drug resistance seen with direct virus-targeting antivirals.[8] Several studies have already demonstrated the feasibility of this approach, such as miR-155 suppression of heterologous nuclear ribonucleoprotein C1/C2, which is critical for cytoplasmic poliovirus replication,[9] and Japanese encephalitis virus inhibition by miR-33a-5p downregulation of eukaryotic translation elongation factor 1A1, which stabilizes the components of the viral replicase complex.[10]

There is an unmet clinical need for novel antiviral therapeutics to treat respiratory virus infection, particularly agents that could be effective against multiple viral strains and in scenarios of co-infection. We have previously identified miRNAs that have broad-spectrum antiviral activity against herpesviruses,[11] and here we present data extending the antiviral profile of a number of these miRNAs against influenza A virus (IAV) and respiratory syncytial virus (RSV). Several miRNAs were identified that cause suppression of viral replication in all respiratory viruses screened. Investigation into the miRNA antiviral mechanism of action identified the p38 mitogen-activated protein kinase

Received 29 September 2016; accepted 28 March 2017;
http://dx.doi.org/10.1016/j.omtn.2017.03.008.

Correspondence: Amy H. Buck, Institute of Immunology and Infection Research and Centre for Immunity, Infection and Evolution, School of Biological Sciences, University of Edinburgh, Charlotte Auerbach Road, Edinburgh EH9 3FL, UK.
E-mail: a.buck@ed.ac.uk

 © 2017 The Authors.
This is an open access article under the CC BY license (http://creativecommons.org/licenses/by/4.0/).

Table 1. The miRNAs Previously Identified as Antiviral against MCMV, MHV-68, and HSV-1

miRNA	Sequence	MCMV Inhibition (%)	MHV-68 Inhibition (%)	HSV-1 Inhibition (%)
miR-24	UGGCUCAGUUCAGCAGGAAC	24	84[a]	74
miR-27b	UUCACAGUGGCUAAGUUCUGC	33	56	52
miR-28	AAGGAGCUCACAGUCUAUUGAG	63[a]	80[a]	81
miR-30a-3p	CUUUCAGUCGGAUGUUUGCAGC	36	52	43
miR-34b	UAGGCAGUGUAAUUAGCUGAU	42[a]	84[a]	50
miR-103	AGCAGCAUUGUACAGGGCUAUGA	60[a]	40	60[a]
miR-107	AGCAGCAUUGUACAGGGCUAUCA	67[a]	35	75
miR-124a	UAAGGCACGCGGUGAAUGC	32	87[a]	46
miR-128a	UCACAGUGAACCGGUCUCUUU	59[a]	43	72
miR-129-5p	CUUUUUGCGGUCUGGGCUUGC	42[a]	62	70[a]
miR-155	UUAAUGCUAAUUGUGAUAGGGGU	43[a]	38	65
miR-199a-3p	UACAGUAGUCUGCACAUUGG	43[a]	76	78[a]
miR-214	ACAGCAGGCACAGACAGGCAGU	47[a]	87[a]	16
miR-222	AGCUACAUCUGGCUACUGGGU	38	54	70
miR-223	UGUCAGUUUGUCAAAUACCCCA	35[a]	51	49
miR-345	GCUGACCCCUAGUCCAGUGCUU	66[a]	87[a]	71
miR-346	UGUCUGCCCGAGUGCCUGCCUCU	66[a]	92[a]	91[a]
miR-452	UGUUUGCAGAGGAAACUGAG	49[a]	62	43
miR-542-5p[b]	CUCGGGGAUCAUCAUGUCA	76[a]	94[a]	86[a]
miR-744	UGCGGGGCUAGGGCUAACAGCA	47[a]	93[a]	75[a]

Percentage inhibition is shown as comparison to average negative transfection control based on suppression of virus using GFP reporter assays, as described in Santhakumar et al.[11]
[a]Antiviral ranking was statistically significant when comparing replicate viral screens ($p < 0.05$).
[b]The mmu-miR-542-5p sequence from miRBase was used in screening which differs by 1 nt from hsa-miR-542-5p.

(MAPK) host pathway as a target of three broad-spectrum miRNAs from distinct miRNA families. Furthermore, we examined p38 MAPK downstream kinases, MAPK-activated protein kinase (MK) 2 and 3 for their importance in IAV and RSV infection. Our results demonstrate that host-targeting antiviral miRNAs could provide a complementary strategy for controlling infection, and they further illuminate host factors that are important in respiratory virus infection.

RESULTS

Screening for Antiviral miRNAs against IAV and RSV

We previously conducted a screen of 312 mouse miRNAs for their effect on herpesvirus infection, and we identified miRNA mimics that had antiviral or proviral activity.[11] Here we further screen a subset of these miRNAs that were selected based on their conservation between mouse and human genomes and the fact that they caused a reduction in viral growth in all three herpesviruses tested (murine cytomegalovirus [MCMV], murine gammaherpesvirus-68 [MHV-68], and herpes simplex virus 1 [HSV-1]) (Table 1). As the genomes of these viruses share little sequence similarity, it was proposed that the impact of the selected miRNAs on viral growth relates to their regulation of host genes, rather than direct interactions with viral elements.

To examine the breadth of these antiviral activities in other viral infections, we screened the miRNA mimic panel against IAV and RSV in human cells (Figure 1). The adenocarcinomic human alveolar basal epithelial cell line A549 was used as these cells are amenable to small RNA transfection and are permissive to the majority of lab-adapted IAV and RSV strains. A549 cells were transfected with 25 nM miRNA mimics 48 hr prior to infection with influenza A/WSN/1933 (WSN) H1N1, and viral titer was assessed at 24 hr post-infection (hpi). Of the 20 miRNAs screened based on their repressive properties in herpesvirus infection, eight also caused significant suppression of IAV WSN when using a cutoff of 75% reduction compared to negative controls (Figure 1A).

Further miRNA mimic screening was conducted with two additional strains of influenza, A/Puerto Rico/8/1934 (PR8) H1N1 (Figure 1B) and A/Udorn/307/1972 (Udorn) H3N2 (Figure 1C). When the same 75% reduction in viral titer cutoff was applied to these screens, six and ten miRNAs were classified as causing suppression of PR8 H1N1 and Udorn H3N2, respectively. This identified five miRNAs with antiviral effects across all three IAV strains as follows: miR-542-5p, miR-24, miR-124, miR-744, and miR-155 (>75% reduction; Table 2).

The same panel of miRNAs was subsequently screened against two different strains of RSV: the prototypic lab-adapted strain RSV-A2 (Figure 1D) and the clinically relevant strain RSV BT2a (Figure 1E).

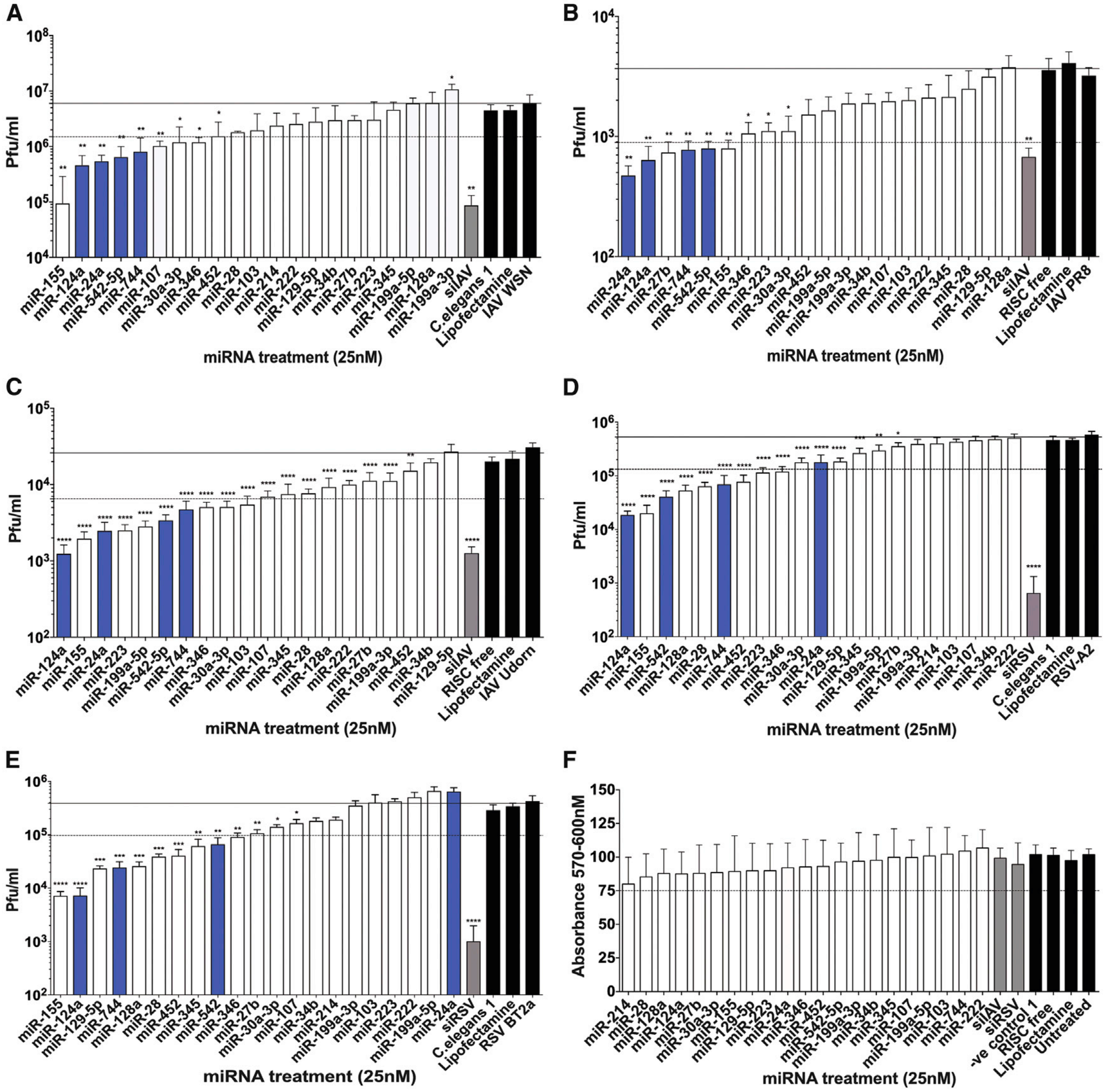

Figure 1. Reductions in IAV and RSV Titer following Treatments with miRNA Mimics

A549 cells were transfected with 25 nM miRNA mimics or controls. A direct-targeting viral siRNA (siIAV or siRSV) served as a positive antiviral control (gray bar), while negative controls were as follows: *C. elegans* miRNA mimic 1 (C.elegans 1), RISC-free siRNA, Lipofectamine, and virus infection alone (black bars). (A–E) After 48-hr transfection, media was removed and cells were infected with (A) IAV WSN H1N1 at MOI 0.1 for 24 hr, (B) IAV PR8 H1N1 at MOI 0.1 for 12 hr, (C) IAV Udorn H3N2 at MOI 0.1 for 24 hr, (D) RSV-A2 at MOI 0.01 for 72 hr, and (E) RSV-BT2a at MOI 0.1 for 72 hr, when the supernatant was removed and assayed for virus. (F) Uninfected cells were analyzed for cell viability at 48 hr post-transfection (n = 12). The results are displayed to show the most potent antiviral mimics (from left to right along the x axis), with solid lines denoting 0% inhibition, dotted lines denoting the 75% activity cutoff, and blue bars highlighting miRNAs chosen for further analysis. Viral titer results for each virus are shown as the mean ± SEM of n = 6. Significant differences between virus control and miRNA mimics and siRNAs are indicated (*p < 0.05, **p < 0.01, ***p < 0.001, and ****p < 0.0001, one-way ANOVA).

There were eight mimics that displayed a >75% reduction in both RSV strains: miR-124a, miR-542-5p, miR-744, miR-155, miR-346, miR-452, miR-128a, and miR-28 (Table 2). Cell viability assays were performed in parallel with the screening experiments, and, consistent with our previous results in the NIH 3T3 mouse embryo fibroblast cell line,[11] none of the panel of 20 miRNAs resulted in cellular toxicity after 48 hr transfection in A549 cells (Figure 1F).

Based on the screening data comparison shown in Table 2, four miRNA mimics, miR-124a, miR-542-5p, miR-744, and miR-155, had broad-spectrum antiviral activity against all IAV and RSV strains tested. Of these four miRNAs, miR-155 has been well documented to regulate innate immunity, enhancing IFN-inducible gene expression.[12,13] Although this miRNA has antiviral properties, we elected to focus on the other miRNAs that may inhibit viral infection without

Table 2. Antiviral Activity of miRNA Panel against IAV and RSV

	Influenza			RSV	
miRNA	H1N1 WSN (%)	H1N1 PR8 (%)	H3N2 Udorn (%)	RSV-A2 (%)	RSV BT2a (%)
miR-124a	92*	82*	95*	96*	98*
miR-542-5p	86*	75*	87*	92*	83*
miR-744	80*	82*	82*	87*	94*
miR-155	98*	78*	92*	96*	98*
miR-24a	92*	91*	90*	66	−68
miR-346	80*	70	80*	77*	76*
miR-452	72	49	41	85*	90*
miR-28	70	29	70	88*	90*
miR-128a	9	11	64	90*	93*
miR-223	54	69	90*	78*	−10
miR-103	80*	43	79*	18	−4
miR-129-5p	65	21	−5	65	94*
miR-107	87*	44	73	12	57
miR-27b	63	83*	57	32	72
miR-345	15	60	71	49	84*
miR-199a-5p	−44	61	89*	43	−72
miR-30a-3p	72	69	80*	66	64
miR-34b	26	55	24	9	53
miR-199a-3p	−93	50	57	25	9
miR-222	42	41	61	3	−31
miR-214	47	Nt	Nt	23	50

Values denote average percentage reduction in viral titer compared to negative control (n = 6). Nt indicates not tested and negative values denote increases in viral titer compared to negative control. The asterisks indicate >75% reduction in viral titer compared to negative control.

stimulating an interferon response. In addition, miR-24 exhibited antiviral activity against all influenza strains tested here. Although we did not observe >75% suppression of RSV, this miRNA was previously shown to be regulated by RSV infection,[14] and it was subsequently chosen for further analysis. Therefore, the following four antiviral miRNA mimics were selected for further analysis of their potential mode of action: miR-542-5p, miR-744, miR-124a, and miR-24.

Antiviral miRNA Mechanism of Action

To gain insight on the host pathways targeted by the four antiviral miRNA mimics, a reverse-phase protein array (RPPA) was used to screen the expression levels of global signaling pathway markers. The advantage of conducting the RPPA instead of a microarray or RNA sequencing (RNA-seq) was that it enabled protein levels to be examined and, in several cases, distinguished the phosphorylation state of proteins, which is often relevant for the activation status of signaling pathways.

Human A549 cells were either left untreated or transfected with miRNA mimics and mock infected or infected with IAV WSN H1N1. Samples were collected at 4 and 24 hpi in order to assess the effects of the miRNAs at both early and late time points. In total, 45 proteins were examined using 60 validated antibodies, 15 of which detected phosphorylated proteins. These antibodies span global signaling pathways associated with metabolism, cell cycle, and immune responses (Table S1), and 59 of the antibodies yielded signal suitable for quantitation (Materials and Methods; Table S2). As a validation of the approach, RPPA proteins were examined for changes of >25% in expression upon infection compared to levels previously reported in the literature (Table S3). As expected, several pathways were activated upon infection, including PKC, which is rapidly activated by influenza hemagglutinin[15] and is critical for enveloped virus entry;[16,17] ERK, which is upregulated by the influenza matrix protein[18] and is essential for viral RNP formation and nuclear export;[19,20] and nuclear factor κB (NF-κB), which has been shown to be crucial for IAV infection[21–23] (Figure 2A, top). In addition, several markers that have previously been shown to be downregulated upon IAV infection were also suppressed in the RPPA study, including JAK1[24–26] and β-tubulin[27,28] (Figure 2A, bottom).

Changes in the global pathway signaling markers caused by miRNA treatment at 24 hpi are shown in Figure 2B, which were normalized to the RISC free non-targeting short interfering (siRNA) transfection control. Overall, the protein expression alterations caused by miR-744, miR-124a, and miR-24 treatment in IAV-infected cells had a higher correlation of similarity than any of these miRNAs with miR-542 (Figure 2B). One pathway that demonstrated commonality among these three miRNAs was suppression of the p38 MAPK pathway. Total p38 MAPK protein expression and its downstream signaling partner MK2 were downregulated by miR-744, miR-124, and miR-24 at 24 hpi (Figures 2C and 2D).

The p38 MAPK Pathway Is Suppressed by miR-744, miR-124a, and miR-24 Mimic Treatment in IAV Infection

It is well documented that the p38 MAPK pathway is activated by various external stimuli, including pro-inflammatory cytokines and viral infection.[29,30] Upon activation of p38 MAPK, the phosphorylated protein regulates downstream kinases MK2 and MK3, mitogen- and stress-activated protein kinases (MSKs) 1 and 2, and several transcription factors, including activating transcription factor 2 (ATF-2), signal transducer and activator of transcription 1 (STAT1), and Myc (Figure 3A). This pathway is known to be upregulated upon influenza virus infection (Figure S1), with the suppression of p38 MAPK[31,32] or the downstream target MK2,[33] causing the attenuation of influenza virus replication. Therefore, it was hypothesized that at least part of the miRNA antiviral activity observed following influenza infection may be attributed to suppression of the p38 MAPK pathway.

To further validate the downregulation of p38 MAPK and MK2 by the miRNA mimics, cells were transfected with miR-744, miR-124a, and miR-24 mimics or inhibitors, and changes in the p38 MAPK pathway were assessed during IAV infection by western blot (Figures 3B–3H). As the RPPA results were similar between the 10 and 25 nM miRNA mimic treatments (Table S2), a concentration of 10 nM was used for

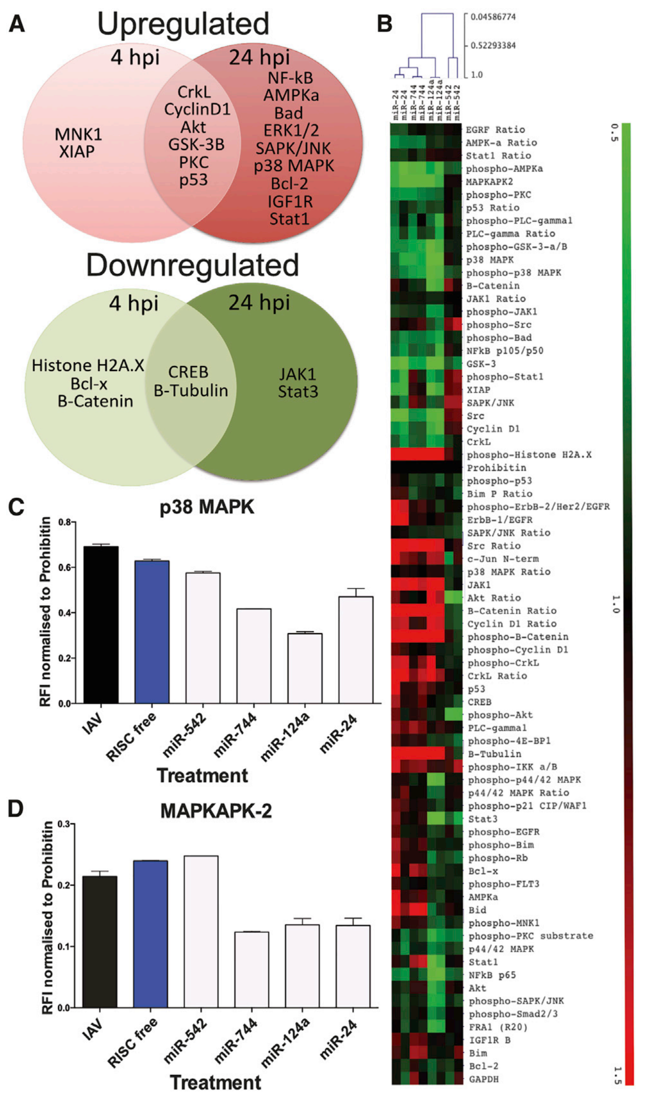

Figure 2. Analysis of Changes in Global Pathway Signaling Molecules upon Infection or miRNA Transfection

(A) Venn diagram of RPPA markers in untransfected cells whose levels were increased or decreased by >25% upon infection when compared to the uninfected average. (B) Cells were transfected with 25 nM miRNA mimics or controls and subsequently infected with IAV WSN at MOI 3. Heatmap shows protein levels analyzed by RPPA at 24 hpi, normalized to RISC-free siRNA treatment. Hierarchical clustering was conducted with Pearson correlation. (C and D) Relative fluorescence intensity of (C) total p-p38 MAPK + p38 MAPK or (D) MAPKAPK2 protein levels from RPPA examination is shown as the mean ± SEM of n = 2.

all further experiments. In agreement with the RPPA results, miR-744, miR-124a, and miR-24 miRNA mimic treatments of IAV-infected cells caused a significant decrease in phosphorylated p38 MAPK (Figure 3C) and total MK2 levels (Figure 3E). Analysis of p38 MAPK levels showed that miR-744, miR-124a, and miR-24 mimic treatments caused a reduction in p38 MAPK expression, with treatment with miR-124a causing a significant decrease in protein levels (Figure 3D). These results demonstrate that miR-744, miR-124a, and miR-24 mimics suppress both p38 MAPK expression and activation. The inhibitors of miR-744, miR-124a, and miR-24 demonstrated unchanging or significant increased expression of phosphorylated p38 MAPK (Figure 3C) and total MK2 levels (Figure 3E).

All three of these miRNAs have been detected in A549 cells by small RNA sequencing, although at relatively low levels compared to more dominant miRNAs in this cell type (GEO: GSM1401418).[34] Consistent with the miRNA mimic treatment decrease in MK2, we also observed a reduction in phosphorylation of heat shock protein 27 (Hsp27), a cytoplasmic substrate of MK2 (Figure 3F). Hsp27 has been shown to mediate cytoskeletal stability, cell motility, apoptosis, and IL-1-induced expression of pro-inflammatory mediators.[35–38] As an effector of MK2 activity, its phosphorylation was investigated to determine whether the reduction of total MK2 levels resulted in downstream downregulation of Hsp27. Activation of Hsp27 was significantly decreased by miR-744 and miR-124a, but not by miR-24 treatment (Figure 3F). In addition, the transcription factor Myc that is activated by p38 MAPK was decreased by miR-744 and miR-124a treatments (Figure 3G). This transcription factor has been shown via chromatin immunoprecipitation sequencing (ChIP-seq) analysis to potentially control MK2 expression, and its downregulation may result in the decrease of MK2 expression.[39] Together these results suggest that, while miR-24, miR-124a, and miR-744 all result in downregulation of the p38 MAPK pathway, these miRNAs may operate by targeting different pathway members to facilitate MK2 suppression.

MK2 Suppression Is Only Partly Responsible for the miRNA Antiviral Activity

To examine whether the antiviral activity of the miRNAs could be explained solely by their effects on MK2, a comparison was conducted between the miRNA mimics and an siRNA directly targeting MK2 (siMK2). As MK3 has been hypothesized to be a homologous kinase of MK2 and share many of the same downstream effectors, the effect of its suppression on IAV replication was also investigated with an siRNA (siMK3).

Changes in MK2 and MK3 expression were assessed by western blot during IAV infection in cells transfected with siRNAs or miRNA mimics (Figure 4A). Consistent with our previous results, treatments with the miR-744, miR-124a, and miR-24 mimics caused a significant reduction in MK2 expression when compared to the negative transfection control (67%, 45%, and 62% reductions, respectively; Figure 4B). As expected, silencing of MK2 caused by the MK2 siRNA (90% reduction) was superior to any of the miRNA mimic treatments (Figure 4B). Analysis of the MK3 protein levels showed that the siMK3 treatment caused a significant decrease in MK3 expression (89% reduction), while miR-542, miR-744, miR-124a, and miR-24 treatments had no significant effect on MK3 levels (Figure 4C).

IAV titer was examined at 24 hpi, and treatment with siMK3 did not cause a significant decrease in IAV titer (Figure 4D), suggesting that

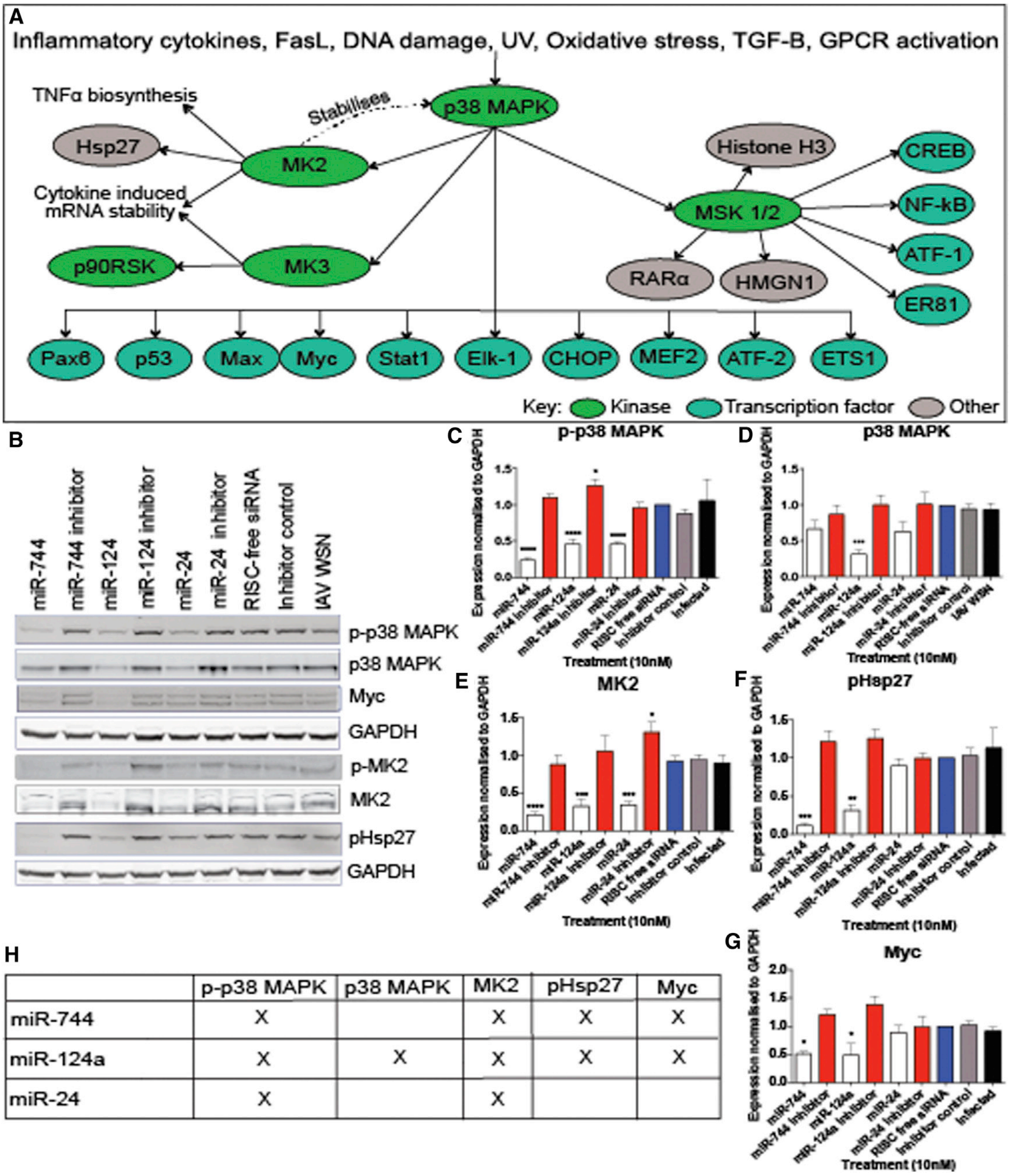

Figure 3. Validation of p38 MAPK Pathway Downregulation by Antiviral miRNA Mimics

(A) p38 MAPK-signaling pathway including kinases, transcription factors, and other genes induced or activated by p38 stimuli. (B) Western blot analysis of p38 MAPK pathway mediators in A549 cells that were transfected with 10 nM miRNA mimics, 25 nM inhibitors, or controls and subsequently infected with IAV WSN at MOI 3 for 24 hr. (C–G) Quantitative analysis of (C) p-p38 MAPK, (D) p38 MAPK, (E) MK2, (F) pHsp27, and (G) Myc total protein levels normalized to GAPDH shown as the mean ± SEM of n = 4. (H) Summary of quantitative analysis with X denoting significant downregulation. Significant differences between RISC-free siRNA control and miRNA mimics and inhibitors are indicated (*p < 0.05, **p < 0.01, ***p < 0.001, and ****p < 0.0001, one-way ANOVA).

this protein does not have an essential function in IAV replication. Conversely, miR-542, miR-744, miR-124a, miR-24, and siMK2 treatments caused a significant decrease in IAV replication. However, while siMK2 treatment resulted in significantly decreased MK2 protein expression when compared to miR-124a treatment (p < 0.01; Figure 4B), it did not have superior antiviral activity (Figure 4D).

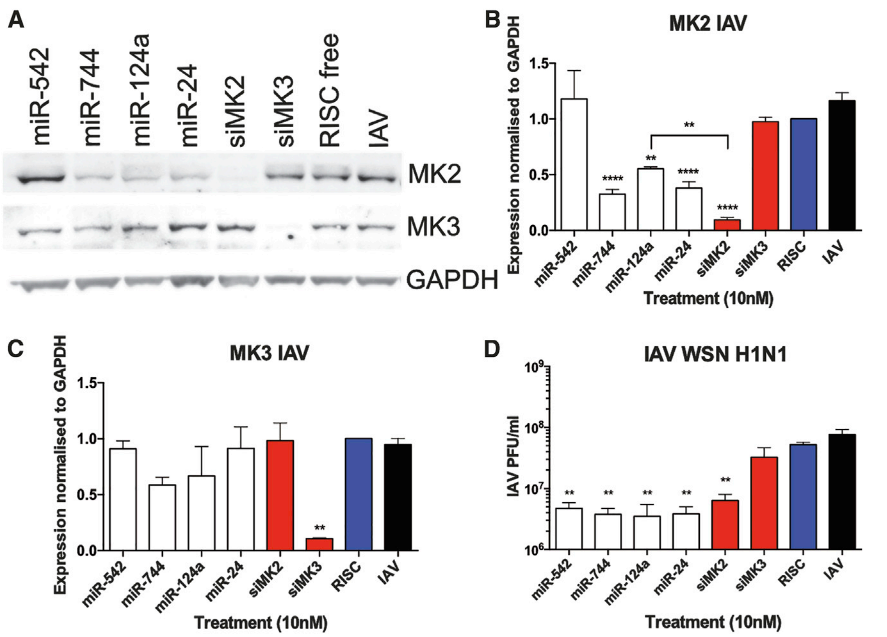

Figure 4. Role of MK2 Suppression in IAV Infection

(A) Representative western blot analysis of MK2 and MK3 expression in A549 cells transfected with 10 nM miRNA mimics, siMK2, siMK3, or controls for 48 hr. Cells were subsequently infected with IAV WSN at MOI 3 for 24 hr. (B and C) Quantitative analysis of (B) MK2 or (C) MK3 total protein levels during IAV infection normalized to GAPDH shown as the mean ± SEM of n = 4. (D) IAV viral titer is shown as the mean ± SEM of n = 4. Significant differences between RISC-free siRNA control and miRNA or siRNA treatments are indicated (*p < 0.05, **p < 0.01, and ****p < 0.0001, one-way ANOVA).

Together these results confirm the previous finding that MK2 suppression is antiviral in the context of IAV infection, and they also suggest that miR-744, miR-124a, and miR-24 antiviral activity is due, at least in part, to MK2 targeting, in addition to concurrent targeting of other proteins important in IAV infection.

MK2 Suppression Is Antiviral for RSV Infection

The importance of p38 MAPK in RSV infection has been previously established, with p38 MAPK inhibitors causing a significant decrease in RSV replication.[32] However, the role of downstream MK2 is less clear, as studies have also shown that RSV sequesters phosphorylated p38 MAPK into cytoplasmic inclusion bodies upon infection, which might suggest that suppression of downstream kinases would be advantageous for the virus.[40] Therefore, an analysis of the potential antiviral properties of direct MK2 suppression in RSV infection was conducted to determine whether this pathway could be responsible for a portion of the antiviral effects shown by miR-744, miR-124a, and miR-24.

Cells were transfected with siRNAs targeting MK2 and MK3 or the miRNA mimics, and MK2 and MK3 expression was assessed during RSV replication by western blot (Figure 5A). In agreement with the results in IAV infection, treatments with miR-744, miR-124a, miR-24, and siMK2 caused a significant decrease in MK2 protein expression (Figure 5B), while treatment with siMK3 significantly decreased the targeted MK3 levels (Figure 5C). While miR-744 and miR-124a mimic treatments both showed a decrease in MK3 expression, these decreases did not reach statistical significance (Figure 5C).

An examination of RSV titers at 24 hpi showed that treatment with siMK3 did not cause a significant reduction of RSV (Figure 5D), indicating that this protein does not have a significant function in RSV replication. Conversely, treatment with the MK2-targeting siRNA siMK2 resulted in a significant reduction of RSV titers (Figure 5D). As seen in IAV infection, while miR-744, miR-124a, and miR-24 did not result in the same level of MK2 protein downregulation when compared to the directly targeting siMK2 (Figure 5B), these miRNAs had equal or superior suppression of RSV replication (Figure 5D).

DISCUSSION

In light of the constant emergence of new respiratory viruses, and the limited availability and efficacy of current vaccines and antiviral drugs against the majority of these, there is a demand for new targeting strategies with broad-spectrum antiviral activity.

We have previously identified a panel of 20 miRNAs that caused a reduction in viral growth of three different herpesviruses (Table 1).[11] In this study, we screened the panel of 20 miRNA mimics against IAV H1N1 WSN, IAV H1N1 PR8, IAV H3N2 Udorn, RSV-A2, and RSV BT2a, and we selected for further analysis one miRNA that had antiviral activity of >75% against all IAV strains tested and three miRNAs that had antiviral activity of >75% against both IAV and RSV (Figure 1). Importantly, none of the panel of 20 miRNAs demonstrated cellular toxicity in A549 (Figure 1F) and NIH 3T3 cells.[11] The four selected miRNAs, miR-24, miR-124a, miR-542, and miR-744, are from unrelated miRNA families and are not known to be co-regulated.[41] While miR-24 and miR-124a were previously shown to have important roles in cardiac, neurological, and oncological diseases,[42–45] the targets of miR-744 and miR-542 have not yet been extensively investigated.

An RPPA analysis provided initial insights into the potential mechanism of action of the antiviral miRNAs during influenza virus infection. The effects of miR-124, miR-24, and miR-744 on protein expression were more closely related than those of miR-542, based on clustering analysis. In particular, treatments with the three former miRNAs regulated the p38 MAPK pathway, causing the downregulation of both p38 MAPK and its downstream kinase

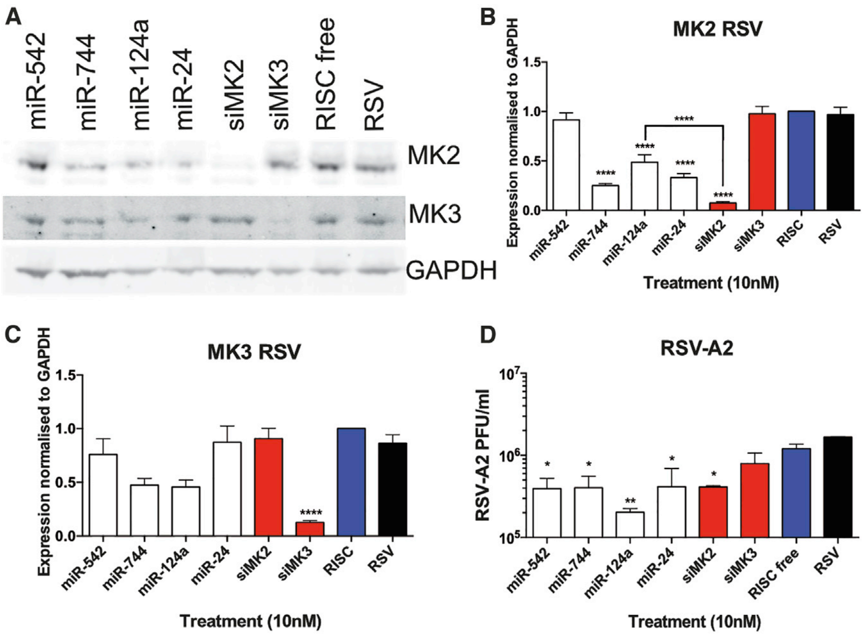

Figure 5. Role of MK2 Suppression in RSV Infection

(A) Representative western blot analysis of MK2 and MK3 expression in A549 cells transfected with 10 nM miRNA mimics, siMK2, siMK3, or controls for 48 hr. Cells were subsequently infected with RSV-A2 at MOI 3 for 24 hr. (B and C) Quantitative analysis of (B) MK2 or (C) MK3 total protein levels in RSV infection normalized to GAPDH shown as the mean ± SEM of n = 4. (D) RSV viral titers are shown as the mean ± SEM of n = 3. Significant differences between RISC-free siRNA control and miRNA or siRNA treatments are indicated (*p < 0.05, **p < 0.01, and ****p < 0.0001, one-way ANOVA).

MK2. Validation of p38 MAPK and MK2 suppression and further analysis of additional pathway factors showed that each miRNA caused a distinct pattern of p38 MAPK pathway downregulation (Figure 3), suggesting they may operate by different factors. Previous research has identified p38α (MAPK14), the dominant form of p38 MAPK, as a direct target of miR-24[46] and miR-124a.[47,48] In addition, as MK2 is known to stabilize p38 MAPK,[49] it could be expected that the significant decrease seen in MK2 by miR-24, miR-124a, and miR-744 treatments may also result in a reduction in p38 MAPK expression. Reciprocally, MK2 levels are also known to be decreased in p38α-deficient cells,[50] which may account for the significant decrease in MK2 expression observed (Figure 3). An analysis of the 3′ UTR of the *mapkapk2* gene via TargetScanHuman[51] revealed target sites for miR-124a and miR-24, suggesting possible direct interactions. An additional mechanism of total MK2 suppression may be via reductions in Myc levels, as previous ChIP-seq analysis of Myc has identified it as a potential transcription factor regulating MK2 expression.[39] Furthermore, Myc is a previously validated target of miR-24[52] and miR-744.[53] Surprisingly, analysis of Myc expression showed that miR-24 treatment did not result in a significant decrease in Myc protein levels (Figure 3). However, miR-744 and miR-124a treatments caused a significant decrease in Myc expression, which may explain the resulting reduction in MK2 expression. As Myc has previously been shown to be a host factor essential for influenza virus replication, a reduction in Myc expression would also be expected to cause virus attenuation.[54]

As mentioned above, the reductions of p38 MAPK and MK2 expression were previously shown to be antiviral in influenza virus infection.[32,33] In agreement with these results, this study found that siRNA silencing of MK2 caused a significant decrease in IAV infection (Figure 4). Subsequent analysis of MK2 silencing during RSV infection showed for the first time that MK2 suppression was also antiviral in the context of RSV infection (Figure 5). Indeed, a downregulation of MK2 may be responsible in part for the broad-spectrum antiviral effects seen by miR-124a, miR-744, and miR-24 treatments. Interestingly, MK2 suppression is also antiviral in adenovirus infection due to the requirement of Hsp27 activation for virus nuclear targeting.[55]

An important discovery from this study was that, while a portion of the broad-spectrum antiviral activity of miR-744, miR-124a, and miR-24 resulted from the suppression of MK2, the entirety of each miRNA antiviral activity could not be attributed to the suppression of this single host factor. The RPPA study identified additional proteins that may be targeted by these miRNAs, such as miR-744 targeting of Akt or miR-124a and miR-24 suppression of GSK-3β, as both of these factors were previously shown to be important for influenza virus entry.[56,57] Other previously identified roles of these miRNAs in viral infection include miR-24 suppression of Kruppel-like factor 6, which is induced by RSV infection to induce cell-cycle arrest,[14] as well as the suppression of furin by miR-24, which is required for influenza virus activation,[58] and miR-124a attenuation of Japanese encephalitis virus (JEV) via targeting dynamin2, which is required for efficient JEV replication.[59] Therefore, it is highly probable that the broad-spectrum antiviral activity exhibited by these miRNA mimics is caused by their ability to downregulate multiple host factors that are essential for numerous DNA and RNA viral infections. A complete understanding of miRNA function may, therefore, require an in depth investigation of the multiple targets that are important in each infection setting. This may also hold true in developing more effective antiviral therapeutics.

In conclusion, this study has extended the broad-spectrum antiviral activity of four miRNA mimics in the context of IAV and RSV infection. For three of these miRNAs, a portion of their antiviral activity was attributed to their suppression of the p38 MAPK pathway and MK2 in particular. This work therefore pinpoints additional host

factors with broad-spectrum antiviral activity, and it demonstrates that MK2 suppression is antiviral in RSV infection. Further studies are required to investigate the effects of miR-124a, miR-24, miR-744, and miR-542 in primary cells and/or an animal model of virus infection and to explore whether their antiviral activity extends to the entire range of viruses that cause respiratory infections and lack treatment strategies.

MATERIALS AND METHODS

Antibodies, miRNAs, and siRNAs

RISC-free (non-targeting) siRNA, murine and *Caenorhabditis elegans* miRNA inhibitors, and mimics were obtained from Dharmacon Products, GE Life Sciences. IAV-[60] and RSV-targeting[61] siRNAs were obtained from QIAGEN, MK2-targeting siRNA was obtained from Sigma-Aldrich, and the MK3-targeting pool of siRNAs was obtained from Dharmacon.

The following primary antibodies were obtained from Cell Signaling Technology: Phospho-p38 MAPK (Thr180/Tyr182, clone 3D7), GAPDH (clone 14C10), c-Myc (clone D84C12), Phospho-CREB (clone 87G3), MK3 (clone D54E4), and Phospho-MK2 (Thr334). Other primary antibodies were as follows: biotinylated anti-RSV antibody (AbD Serotec), Phospho-HSP27 (S86, Abcam), and MK2 (clone E341, Abcam). Secondary antibodies were Alexa Fluor 680-conjugated anti-rabbit IgG antibody (Invitrogen) and Alexa Fluor 800-conjugated anti-mouse IgG antibody (Invitrogen).

Cell Culture and Viruses

A549 human lung epithelial cells (ATCC), Madin-Darby canine kidney (MDCK) cells (ATCC), and human epithelial type 2 (HEp-2) cells were cultured in DMEM (Sigma) supplemented with 10% heat-inactivated fetal bovine serum (FBS; Hyclone, GE Healthcare) and 1% L-Glutamine (Gibco, Life Technologies) at 37°C and 5% CO_2. All cells were confirmed to be free of mycoplasma via PCR detection with primers (forward: 5′-GGGAGCAAACAGGATTAGATACCC-3′ and reverse: 5′-TGCACCATCTGTCACTCTGTTAACCTC-3′), as previously described.[62]

Viruses used in the study were influenza A strains A/Puerto Rico/8/1934 H1N1 (PR8), A/WSN/1933 H1N1 (WSN), and A/Udorn/307/1972 H3N2 (Udorn) as well as RSV-A2 (VR-1540, ATCC) and RSV clinical strain BT2a.[63]

Transfection

The miRNA mimics or inhibitors were reverse-transfected into A549 cells in 0.3% Lipofectamine 2000 (Invitrogen). Transfected cells were incubated for 48 hr at 37°C and 5% CO_2 before cell viability analysis or viral challenge.

Infection Assays

Cells were infected by the addition of virus in a minimal volume at the appropriate dilution to give the indicated MOI for 1 hr at 37°C. After 1 hr cells were washed with PBS and replenished with fresh media without virus. Cells used to study IAV were cultured post-infection in DMEM, and cells used to study RSV were cultured post-infection in DMEM supplemented with 5% FBS. Supernatants were harvested at the indicated time post-infection and the viral titers were determined by plaque assay.

IAV plaque assays were performed with 10-fold serial dilutions of the virus samples on a confluent monolayer of MDCK cells,[64] overlaid with MEM agarose overlay media containing 0.5% BSA (Fraction V, Fisher Scientific) and 1 μg/ml N-acetyl trypsin. Cells were incubated for 72 hr, and then plaques were fixed and visualized by staining with 0.1% toluidine blue O (Sigma). RSV immuno-plaque assays were performed with 2-fold serial dilutions of the virus samples on a confluent monolayer of HEp-2 cells. Cells were incubated for 24 hr in DMEM, and then they were probed with 1:200 biotinylated RSV antibody and stained with 1:500 ExtrAvidin Peroxidase, with visualization of plaques by the addition of 3-Amino-9-EthylCarbazole (AEC) substrate.

Cell Viability Assay

Following reverse transfection, the effect of miRNAs on cell viability was assessed using the cell titer blue assay (Promega) as per the manufacturer's instructions.

RPPA

Protein concentrations were determined by Coomassie-plus assay (Thermo Scientific), and samples were diluted with buffer CSBL1 to 0.15 mg/mL. The 60 validated signaling pathway markers listed in Table S1 were profiled in a standard ZeptoMARK, as previously described.[65] Briefly, analysis of raw excitation light intensity data was conducted by normalizing the net signal intensity of each sample. For each lysate sample represented by a total of four spots at four dilutions, a mean referenced fluorescence intensity (RFI value) was calculated based on a weighted linear fit through the four normalized sample spots and markers, with negative RFI values excluded. These RFI values were subsequently normalized against Prohibitin housekeeping protein prior to the comparison of analytes across the entire sample series. For heatmap analysis, each treatment group was compared to the normalized RFI values of RISC-free siRNA control group.

Western Blot

Cells were lysed with cell lysis buffer (300 mM NaCl, 50 mM Tris-HCl [pH 7.4], and 0.5% Triton X-100) containing protease (cOmplete, Roche) and phosphatase inhibitors (PhosStop, Roche), spun, and supernatant was collected. Total cell lysates were separated by SDS-PAGE and transferred to Immobilon-FL membranes (Millipore) using a Trans-Blot System (Bio-Rad). Membranes were blocked in Tris-buffered saline containing 0.5% Tween 20 and 5% BSA (Fraction V, Fisher Scientific) for 2 hr at room temperature. Primary antibody binding was achieved overnight at 4°C, whereas far-red fluorescent secondary antibody binding was achieved in 1 hr at room temperature. Odyssey (LI-COR Biosciences) was used for visualization.

Statistical Analysis

Statistics data are expressed as means ± SEM. Groups were compared using one-way ANOVA; p values of <0.05 were considered significant.

SUPPLEMENTAL INFORMATION

Supplemental Information includes one figure and three tables and can be found with this article online at http://dx.doi.org/10.1016/j.omtn.2017.03.008.

AUTHOR CONTRIBUTIONS

J.L.M., A.A., B.M.D., J.S., and A.H.B. conceived and designed the experiments. J.L.M., S.R., and A.A. performed the experiments. J.L.M., S.R., A.A., and J.R. analyzed the data. U.F.P. contributed reagents, materials, and analysis tools. J.L.M. and A.H.B. wrote the paper. All authors have reviewed and agreed to the final version of the manuscript.

CONFLICTS OF INTEREST

The authors declare no conflicts of interest.

ACKNOWLEDGMENTS

We thank Gillian Coakley and Marissa Lear for expert technical assistance and Diwakar Santhakumar for work in previous screening analyses. We thank Neil Carragher and Kenneth Macleod, who performed RPPA studies at the Cancer Research UK Edinburgh Centre RPPA facility, University of Edinburgh. This work was supported by the Scottish Enterprise proof of concept grant PS7305CA21, BBSRC grant BB/J001279/1, and Wellcome Trust-University of Edinburgh Institutional Strategic Support Fund.

REFERENCES

1. Mendell, J.T., and Olson, E.N. (2012). MicroRNAs in stress signaling and human disease. Cell *148*, 1172–1187.

2. Bartel, D.P. (2009). MicroRNAs: target recognition and regulatory functions. Cell *136*, 215–233.

3. Jopling, C.L., Schütz, S., and Sarnow, P. (2008). Position-dependent function for a tandem microRNA miR-122-binding site located in the hepatitis C virus RNA genome. Cell Host Microbe *4*, 77–85.

4. Jopling, C.L., Yi, M., Lancaster, A.M., Lemon, S.M., and Sarnow, P. (2005). Modulation of hepatitis C virus RNA abundance by a liver-specific MicroRNA. Science *309*, 1577–1581.

5. Machlin, E.S., Sarnow, P., and Sagan, S.M. (2011). Masking the 5′ terminal nucleotides of the hepatitis C virus genome by an unconventional microRNA-target RNA complex. Proc. Natl. Acad. Sci. USA *108*, 3193–3198.

6. Trobaugh, D.W., Gardner, C.L., Sun, C., Haddow, A.D., Wang, E., Chapnik, E., Mildner, A., Weaver, S.C., Ryman, K.D., and Klimstra, W.B. (2014). RNA viruses can hijack vertebrate microRNAs to suppress innate immunity. Nature *506*, 245–248.

7. McCaskill, J., Praihirunkit, P., Sharp, P., and Buck, A.H. (2015). RNA-mediated degradation of microRNAs: a widespread viral strategy? RNA Biol. *12*, 579–585.

8. Zumla, A., Rao, M., Wallis, R.S., Kaufmann, S.H., Rustomjee, R., Mwaba, P., Vilaplana, C., Yeboah-Manu, D., Chakaya, J., Ippolito, G., et al.; Host-Directed Therapies Network consortium (2016). Host-directed therapies for infectious diseases: current status, recent progress, and future prospects. Lancet Infect. Dis. *16*, e47–e63.

9. Shim, B.S., Wu, W., Kyriakis, C.S., Bakre, A., Jorquera, P.A., Perwitasari, O., and Tripp, R.A. (2016). MicroRNA-555 has potent antiviral properties against poliovirus. J. Gen. Virol. *97*, 659–668.

10. Chen, Z., Ye, J., Ashraf, U., Li, Y., Wei, S., Wan, S., Zohaib, A., Song, Y., Chen, H., and Cao, S. (2016). MicroRNA-33a-5p Modulates Japanese Encephalitis Virus Replication by Targeting Eukaryotic Translation Elongation Factor 1A1. J. Virol. *90*, 3722–3734.

11. Santhakumar, D., Forster, T., Laqtom, N.N., Fragkoudis, R., Dickinson, P., Abreu-Goodger, C., Manakov, S.A., Choudhury, N.R., Griffiths, S.J., Vermeulen, A., et al. (2010). Combined agonist-antagonist genome-wide functional screening identifies broadly active antiviral microRNAs. Proc. Natl. Acad. Sci. USA *107*, 13830–13835.

12. Su, C., Hou, Z., Zhang, C., Tian, Z., and Zhang, J. (2011). Ectopic expression of microRNA-155 enhances innate antiviral immunity against HBV infection in human hepatoma cells. Virol. J. *8*, 354.

13. Wang, P., Hou, J., Lin, L., Wang, C., Liu, X., Li, D., Ma, F., Wang, Z., and Cao, X. (2010). Inducible microRNA-155 feedback promotes type I IFN signaling in antiviral innate immunity by targeting suppressor of cytokine signaling 1. J. Immunol. *185*, 6226–6233.

14. Bakre, A., Wu, W., Hiscox, J., Spann, K., Teng, M.N., and Tripp, R.A. (2015). Human respiratory syncytial virus non-structural protein NS1 modifies miR-24 expression via transforming growth factor-β. J. Gen. Virol. *96*, 3179–3191.

15. Constantinescu, S.N., Cernescu, C.D., and Popescu, L.M. (1991). Effects of protein kinase C inhibitors on viral entry and infectivity. FEBS Lett. *292*, 31–33.

16. Arora, D.J., and Gasse, N. (1998). Influenza virus hemagglutinin stimulates the protein kinase C activity of human polymorphonuclear leucocytes. Arch. Virol. *143*, 2029–2037.

17. Kunzelmann, K., Beesley, A.H., King, N.J., Karupiah, G., Young, J.A., and Cook, D.I. (2000). Influenza virus inhibits amiloride-sensitive Na+ channels in respiratory epithelia. Proc. Natl. Acad. Sci. USA *97*, 10282–10287.

18. Huang, X., Liu, T., Muller, J., Levandowski, R.A., and Ye, Z. (2001). Effect of influenza virus matrix protein and viral RNA on ribonucleoprotein formation and nuclear export. Virology *287*, 405–416.

19. Ludwig, S., Wolff, T., Ehrhardt, C., Wurzer, W.J., Reinhardt, J., Planz, O., and Pleschka, S. (2004). MEK inhibition impairs influenza B virus propagation without emergence of resistant variants. FEBS Lett. *561*, 37–43.

20. Pleschka, S., Wolff, T., Ehrhardt, C., Hobom, G., Planz, O., Rapp, U.R., and Ludwig, S. (2001). Influenza virus propagation is impaired by inhibition of the Raf/MEK/ERK signalling cascade. Nat. Cell Biol. *3*, 301–305.

21. Nimmerjahn, F., Dudziak, D., Dirmeier, U., Hobom, G., Riedel, A., Schlee, M., Staudt, L.M., Rosenwald, A., Behrends, U., Bornkamm, G.W., and Mautner, J. (2004). Active NF-kappaB signalling is a prerequisite for influenza virus infection. J. Gen. Virol. *85*, 2347–2356.

22. Diebold, S.S., Kaisho, T., Hemmi, H., Akira, S., and Reis e Sousa, C. (2004). Innate antiviral responses by means of TLR7-mediated recognition of single-stranded RNA. Science *303*, 1529–1531.

23. Lund, J.M., Alexopoulou, L., Sato, A., Karow, M., Adams, N.C., Gale, N.W., Iwasaki, A., and Flavell, R.A. (2004). Recognition of single-stranded RNA viruses by Toll-like receptor 7. Proc. Natl. Acad. Sci. USA *101*, 5598–5603.

24. Jia, D., Rahbar, R., Chan, R.W., Lee, S.M., Chan, M.C., Wang, B.X., Baker, D.P., Sun, B., Peiris, J.S., Nicholls, J.M., and Fish, E.N. (2010). Influenza virus non-structural protein 1 (NS1) disrupts interferon signaling. PLoS ONE *5*, e13927.

25. Seo, S.U., Kwon, H.J., Song, J.H., Byun, Y.H., Seong, B.L., Kawai, T., Akira, S., and Kweon, M.N. (2010). MyD88 signaling is indispensable for primary influenza A virus infection but dispensable for secondary infection. J. Virol. *84*, 12713–12722.

26. Uetani, K., Hiroi, M., Meguro, T., Ogawa, H., Kamisako, T., Ohmori, Y., and Erzurum, S.C. (2008). Influenza A virus abrogates IFN-gamma response in respiratory epithelial cells by disruption of the Jak/Stat pathway. Eur. J. Immunol. *38*, 1559–1573.

27. Beloso, A., Martínez, C., Valcárcel, J., Santarén, J.F., and Ortín, J. (1992). Degradation of cellular mRNA during influenza virus infection: its possible role in protein synthesis shutoff. J. Gen. Virol. *73*, 575–581.

28. Han, X., Li, Z., Chen, H., Wang, H., Mei, L., Wu, S., Zhang, T., Liu, B., and Lin, X. (2012). Influenza virus A/Beijing/501/2009(H1N1) NS1 interacts with β-tubulin and induces disruption of the microtubule network and apoptosis on A549 cells. PLoS ONE *7*, e48340.

29. Raingeaud, J., Gupta, S., Rogers, J.S., Dickens, M., Han, J., Ulevitch, R.J., and Davis, R.J. (1995). Pro-inflammatory cytokines and environmental stress cause p38 mitogen-activated protein kinase activation by dual phosphorylation on tyrosine and threonine. J. Biol. Chem. *270*, 7420–7426.

30. Rouse, J., Cohen, P., Trigon, S., Morange, M., Alonso-Llamazares, A., Zamanillo, D., Hunt, T., and Nebreda, A.R. (1994). A novel kinase cascade triggered by stress and heat shock that stimulates MAPKAP kinase-2 and phosphorylation of the small heat shock proteins. Cell *78*, 1027–1037.

31. Börgeling, Y., Schmolke, M., Viemann, D., Nordhoff, C., Roth, J., and Ludwig, S. (2014). Inhibition of p38 mitogen-activated protein kinase impairs influenza virus-induced primary and secondary host gene responses and protects mice from lethal H5N1 infection. J. Biol. Chem. *289*, 13–27.

32. Marchant, D., Singhera, G.K., Utokaparch, S., Hackett, T.L., Boyd, J.H., Luo, Z., Si, X., Dorscheid, D.R., McManus, B.M., and Hegele, R.G. (2010). Toll-like receptor 4-mediated activation of p38 mitogen-activated protein kinase is a determinant of respiratory virus entry and tropism. J. Virol. *84*, 11359–11373.

33. Luig, C., Köther, K., Dudek, S.E., Gaestel, M., Hiscott, J., Wixler, V., and Ludwig, S. (2010). MAP kinase-activated protein kinases 2 and 3 are required for influenza A virus propagation and act via inhibition of PKR. FASEB J. *24*, 4068–4077.

34. Flores, O., Kennedy, E.M., Skalsky, R.L., and Cullen, B.R. (2014). Differential RISC association of endogenous human microRNAs predicts their inhibitory potential. Nucleic Acids Res. *42*, 4629–4639.

35. Huot, J., Houle, F., Rousseau, S., Deschesnes, R.G., Shah, G.M., and Landry, J. (1998). SAPK2/p38-dependent F-actin reorganization regulates early membrane blebbing during stress-induced apoptosis. J. Cell Biol. *143*, 1361–1373.

36. Rousseau, S., Houle, F., Landry, J., and Huot, J. (1997). p38 MAP kinase activation by vascular endothelial growth factor mediates actin reorganization and cell migration in human endothelial cells. Oncogene *15*, 2169–2177.

37. Rane, M.J., Pan, Y., Singh, S., Powell, D.W., Wu, R., Cummins, T., Chen, Q., McLeish, K.R., and Klein, J.B. (2003). Heat shock protein 27 controls apoptosis by regulating Akt activation. J. Biol. Chem. *278*, 27828–27835.

38. Alford, K.A., Glennie, S., Turrell, B.R., Rawlinson, L., Saklatvala, J., and Dean, J.L. (2007). Heat shock protein 27 functions in inflammatory gene expression and transforming growth factor-beta-activated kinase-1 (TAK1)-mediated signaling. J. Biol. Chem. *282*, 6232–6241.

39. Consortium, E.P.; ENCODE Project Consortium (2012). An integrated encyclopedia of DNA elements in the human genome. Nature *489*, 57–74.

40. Fricke, J., Koo, L.Y., Brown, C.R., and Collins, P.L. (2013). p38 and OGT sequestration into viral inclusion bodies in cells infected with human respiratory syncytial virus suppresses MK2 activities and stress granule assembly. J. Virol. *87*, 1333–1347.

41. Kozomara, A., and Griffiths-Jones, S. (2014). miRBase: annotating high confidence microRNAs using deep sequencing data. Nucleic Acids Res. *42*, D68–D73.

42. Cao, Q., Li, Y.Y., He, W.F., Zhang, Z.Z., Zhou, Q., Liu, X., Shen, Y., and Huang, T.T. (2013). Interplay between microRNAs and the STAT3 signaling pathway in human cancers. Physiol. Genomics *45*, 1206–1214.

43. Bienertova-Vasku, J., Novak, J., and Vasku, A. (2015). MicroRNAs in pulmonary arterial hypertension: pathogenesis, diagnosis and treatment. J. Am. Soc. Hypertens. *9*, 221–234.

44. Katoh, M. (2014). Cardio-miRNAs and onco-miRNAs: circulating miRNA-based diagnostics for non-cancerous and cancerous diseases. Front. Cell Dev. Biol. *2*, 61.

45. Volný, O., Kašičková, L., Coufalová, D., Cimflová, P., and Novák, J. (2015). microRNAs in Cerebrovascular Disease. Adv. Exp. Med. Biol. *888*, 155–195.

46. Kiriakidou, M., Nelson, P.T., Kouranov, A., Fitziev, P., Bouyioukos, C., Mourelatos, Z., and Hatzigeorgiou, A. (2004). A combined computational-experimental approach predicts human microRNA targets. Genes Dev. *18*, 1165–1178.

47. Lawson, S.K., Dobrikova, E.Y., Shveygert, M., and Gromeier, M. (2013). p38α mitogen-activated protein kinase depletion and repression of signal transduction to translation machinery by miR-124 and -128 in neurons. Mol. Cell. Biol. *33*, 127–135.

48. Krek, A., Grün, D., Poy, M.N., Wolf, R., Rosenberg, L., Epstein, E.J., MacMenamin, P., da Piedade, I., Gunsalus, K.C., Stoffel, M., and Rajewsky, N. (2005). Combinatorial microRNA target predictions. Nat. Genet. *37*, 495–500.

49. Ronkina, N., Kotlyarov, A., Dittrich-Breiholz, O., Kracht, M., Hitti, E., Milarski, K., Askew, R., Marusic, S., Lin, L.L., Gaestel, M., and Telliez, J.B. (2007). The mitogen-activated protein kinase (MAPK)-activated protein kinases MK2 and MK3 cooperate in stimulation of tumor necrosis factor biosynthesis and stabilization of p38 MAPK. Mol. Cell. Biol. *27*, 170–181.

50. Sudo, T., Kawai, K., Matsuzaki, H., and Osada, H. (2005). p38 mitogen-activated protein kinase plays a key role in regulating MAPKAPK2 expression. Biochem. Biophys. Res. Commun. *337*, 415–421.

51. Agarwal, V., Bell, G.W., Nam, J.W., and Bartel, D.P. (2015). Predicting effective microRNA target sites in mammalian mRNAs. eLife *4*, e05005.

52. Lal, A., Navarro, F., Maher, C.A., Maliszewski, L.E., Yan, N., O'Day, E., Chowdhury, D., Dykxhoorn, D.M., Tsai, P., Hofmann, O., et al. (2009). miR-24 Inhibits cell proliferation by targeting E2F2, MYC, and other cell-cycle genes via binding to "seedless" 3'UTR microRNA recognition elements. Mol. Cell *35*, 610–625.

53. Lin, F., Ding, R., Zheng, S., Xing, D., Hong, W., Zhou, Z., and Shen, J. (2014). Decrease expression of microRNA-744 promotes cell proliferation by targeting c-Myc in human hepatocellular carcinoma. Cancer Cell Int. *14*, 58.

54. Karlas, A., Machuy, N., Shin, Y., Pleissner, K.P., Artarini, A., Heuer, D., Becker, D., Khalil, H., Ogilvie, L.A., Hess, S., et al. (2010). Genome-wide RNAi screen identifies human host factors crucial for influenza virus replication. Nature *463*, 818–822.

55. Suomalainen, M., Nakano, M.Y., Boucke, K., Keller, S., and Greber, U.F. (2001). Adenovirus-activated PKA and p38/MAPK pathways boost microtubule-mediated nuclear targeting of virus. EMBO J. *20*, 1310–1319.

56. Hirata, N., Suizu, F., Matsuda-Lennikov, M., Edamura, T., Bala, J., and Noguchi, M. (2014). Inhibition of Akt kinase activity suppresses entry and replication of influenza virus. Biochem. Biophys. Res. Commun. *450*, 891–898.

57. König, R., Stertz, S., Zhou, Y., Inoue, A., Hoffmann, H.H., Bhattacharyya, S., Alamares, J.G., Tscherne, D.M., Ortigoza, M.B., Liang, Y., et al. (2010). Human host factors required for influenza virus replication. Nature *463*, 813–817.

58. Loveday, E.K., Diederich, S., Pasick, J., and Jean, F. (2015). Human microRNA-24 modulates highly pathogenic avian-origin H5N1 influenza A virus infection in A549 cells by targeting secretory pathway furin. J. Gen. Virol. *96*, 30–39.

59. Yang, S., Pei, Y., Li, X., Zhao, S., Zhu, M., and Zhao, A. (2016). miR-124 attenuates Japanese encephalitis virus replication by targeting DNM2. Virol. J. *13*, 105.

60. Ge, Q., McManus, M.T., Nguyen, T., Shen, C.H., Sharp, P.A., Eisen, H.N., and Chen, J. (2003). RNA interference of influenza virus production by directly targeting mRNA for degradation and indirectly inhibiting all viral RNA transcription. Proc. Natl. Acad. Sci. USA *100*, 2718–2723.

61. DeVincenzo, J., Lambkin-Williams, R., Wilkinson, T., Cehelsky, J., Nochur, S., Walsh, E., Meyers, R., Gollob, J., and Vaishnaw, A. (2010). A randomized, double-blind, placebo-controlled study of an RNAi-based therapy directed against respiratory syncytial virus. Proc. Natl. Acad. Sci. USA *107*, 8800–8805.

62. Ossewaarde, J.M., de Vries, A., Bestebroer, T., and Angulo, A.F. (1996). Application of a Mycoplasma group-specific PCR for monitoring decontamination of Mycoplasma-infected Chlamydia sp. strains. Appl. Environ. Microbiol. *62*, 328–331.

63. Villenave, R., O'Donoghue, D., Thavagnanam, S., Touzelet, O., Skibinski, G., Heaney, L.G., McKaigue, J.P., Coyle, P.V., Shields, M.D., and Power, U.F. (2011). Differential cytopathogenesis of respiratory syncytial virus prototypic and clinical isolates in primary pediatric bronchial epithelial cells. Virol. J. *8*, 43.

64. Cottey, R., Rowe, C.A., and Bender, B.S. (2001). Influenza virus. Curr. Protoc. Immunol. *Chapter 19*, 11.

65. van Oostrum, J., Calonder, C., Rechsteiner, D., Ehrat, M., Mestan, J., Fabbro, D., and Voshol, H. (2009). Tracing pathway activities with kinase inhibitors and reverse phase protein arrays. Proteomics Clin. Appl. *3*, 412–422.

OMTN, Volume 7

Supplemental Information

Broad-Spectrum Inhibition of Respiratory Virus Infection by MicroRNA Mimics Targeting p38 MAPK Signaling

Jana L. McCaskill, Sarah Ressel, Andreas Alber, Jane Redford, Ultan F. Power, Jürgen Schwarze, Bernadette M. Dutia, and Amy H. Buck

Supplementary Table 1. Validated signalling pathway markers analysed via RPPA

Antibody	Supplier	Reference Number	Raised	Dilution
IKK alpha/beta P Ser176/Ser177	Cell Signaling Technologies	2078	rabbit	250
Bim	Epitomics	1036	rabbit	250
ErbB-2/Her2/EGFR P Tyr1248/	Cell Signaling Technologies	2244	rabbit	250
NFkB p65 Ser536	Cell Signaling Technologies	3033	rabbit	250
PLC-gamma1 P Tyr783	Cell Signaling Technologies	2821	rabbit	250
AMPK alpha	Cell Signaling Technologies	2532	rabbit	250
c-Jun N-term	Epitomics	1254-1	rabbit	250
CREB	Cell Signaling Technologies	9197	rabbit	250
Bad P Ser112	Cell Signaling Technologies	9291	rabbit	500
CrkL P Tyr207	Cell Signaling Technologies	3181	rabbit	500
FLT3 P Tyr591 P Tyr591	Cell Signaling Technologies	3461	rabbit	500
JAK1	Cell Signaling Technologies	3332	rabbit	500
MNK1 (MKNK) P Thr197,Thr20	Cell Signaling Technologies	2111	rabbit	500
p44/42 MAPK (ERK1/2)	Cell Signaling Technologies	9102	rabbit	500
p44/42 MAPK (ERK1/2) P Thr2	Cell Signaling Technologies	4370	rabbit	500
Src	Cell Signaling Technologies	2109	rabbit	500
Akt	Cell Signaling Technologies	9272	rabbit	500
Akt P Ser473	Cell Signaling Technologies	4060	rabbit	500
4E-BP1 P Ser65	Cell Signaling Technologies	9451	rabbit	500
beta-Catenin	Cell Signaling Technologies	9562	rabbit	500
SAPK/JNK	Cell Signaling Technologies	9258	rabbit	500
GSK-3-alpha/beta P Ser21/Ser9	Cell Signaling Technologies	9331	rabbit	500
p53 P Ser15	Cell Signaling Technologies	9284	rabbit	500
p38 MAPK PThr180,Tyr182	Cell Signaling Technologies	9211	rabbit	500
p38 MAPK	Cell Signaling Technologies	9212	rabbit	500
SAPK/JNK P Thr183,Tyr185	Cell Signaling Technologies	4668	rabbit	500
Rb P Ser780	Cell Signaling Technologies	9307	rabbit	500
Stat1 P Tyr701	Cell Signaling Technologies	9171	rabbit	500
Src (family) P Tyr416	Cell Signaling Technologies	2101	rabbit	500
Smad2/3 P Ser465/Ser423,Ser467	Cell Signaling Technologies	9510	rabbit	500
Cyclin D1 P Thr286	Cell Signaling Technologies	3300	rabbit	500
Bcl-2	Epitomics	1017-1	rabbit	500
Bid	Epitomics	1008	rabbit	500
Bim P Ser69	Cell Signaling Technologies	4585	rabbit	500
GSK-3-beta	Cell Signaling Technologies	9315	rabbit	500
PLC-gamma1	Cell Signaling Technologies	2822	rabbit	500
MAPKAPK-2	Epitomics	1497-1	rabbit	500
Stat3	Cell Signaling Technologies	9132	rabbit	500
XIAP	Cell Signaling Technologies	2045	rabbit	500
FRA1 (R20)	Santa Cruz	sc-605	rabbit	500
AMPK alpha P Thr172	Cell Signaling Technologies	2535	rabbit	500
IGF1R B	Cell Signaling Technologies	3027	rabbit	500
PKC (pan) P Ser660 (beta-2)	Cell Signaling Technologies	9371	rabbit	1000

PKC substrate P (R/K)X(S*)(Hyd)	Cell Signaling Technologies	2261	rabbit	1000
ErbB-1/EGFR	Cell Signaling Technologies	2232	rabbit	1000
EGFR P Tyr1173	Cell Signaling Technologies	4407	rabbit	1000
beta-Catenin P Ser33,Ser37,Thr41	Cell Signaling Technologies	9561	rabbit	1000
Bcl-x	Epitomics	1018	rabbit	1000
NFkB p105/p50	GeneTex	GTX110585	rabbit	1000
p21 CIP/WAF1 p Thr145	Santa Cruz	20220-R	rabbit	1000
JAK1 P Tyr1022,Thr1023	Invitrogen (Biosource)	44-422G	rabbit	5000
beta-Tubulin	Abcam	ab6046	rabbit	5000
Prohibitin	Santa Cruz	sc-28259	rabbit	5000
p53	Cell Signaling Technologies	9282	rabbit	10000
Histone H2A.X P Ser139	Millipore (Upstate)	05-636	mouse IgG1	250
CrkL	Cell Signaling Technologies	3182	mouse IgG1	500
Stat1	Cell Signaling Technologies	9176	mouse IgG1	500
Cyclin D1	Cell Signaling Technologies	2926	mouse IgG2a	500
PKR	Santa Cruz	sc-6282	mouse IgG2a	500
GAPDH	Abcam	ab9484	mouse IgG2b	2000
rabbit			rabbit	250
mouse IgG1			mouse IgG1	250
mouse IgG2a			mouse IgG2a	500
mouse IgG2b			mouse IgG2b	500

Supplementary Table 2. Mean Referenced Fluorescence Intensity (RFI value) normalised to prohibitin housekeeping gene

Excel file supplied

Supplementary Table 3. Infected sample RFI values normalised to prohibitin and analysed for expression increase or decrease of 25% when compared to the uninfected control average.

Excel file supplied

Supplementary Figure 1.

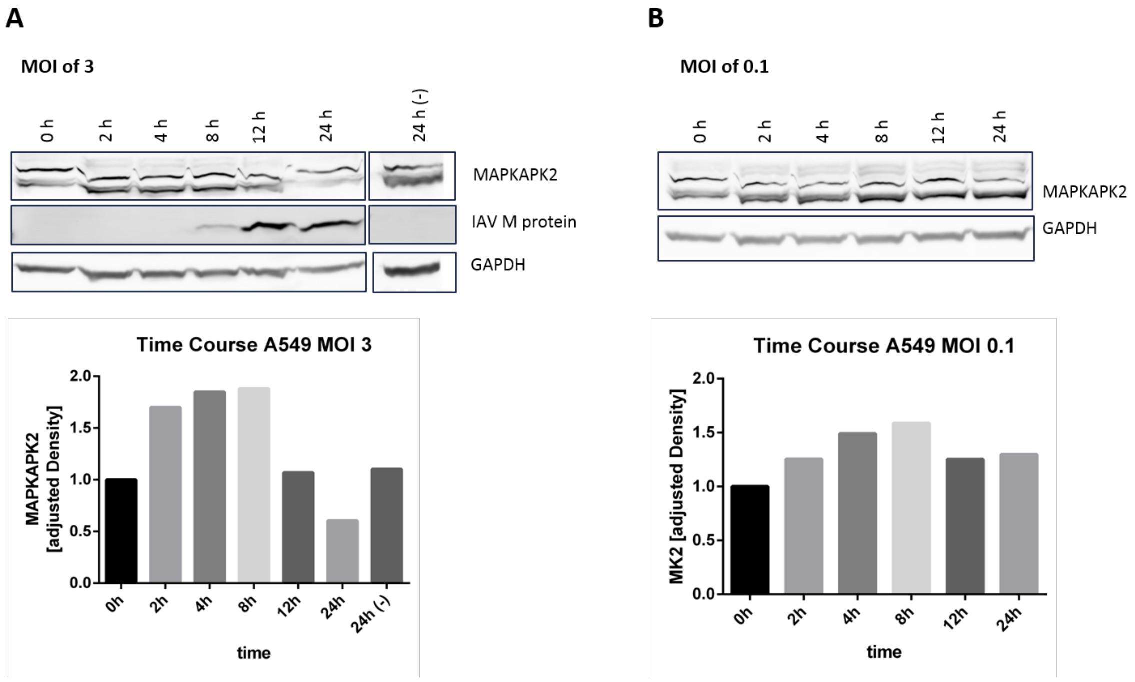

Supplementary Figure 1. Time course of MK2 expression during IAV infection. A549 cells were infected with IAV H1N1 WSN at an **A)** MOI of 3 or a **B)** MOI of 0.1. Western blot analysis and the corresponding band density analysis are displayed for each MOI. Quantitative analysis of MK2 was normalised to the GAPDH loading control and subsequently compared to uninfected sample (0hrs).
